# Glioma-associated microglia/macrophages (GAMs) in glioblastoma: Immune function in the tumor microenvironment and implications for immunotherapy

**DOI:** 10.3389/fimmu.2023.1123853

**Published:** 2023-03-09

**Authors:** Chao Lin, Ning Wang, Chengyan Xu

**Affiliations:** Department of Neurosurgery, Children’s Hospital, Zhejiang University School of Medicine, National Clinical Research Center For Child Health, Hangzhou, Zhejiang, China

**Keywords:** glioma, glioblastoma, glioma-associated macrophage and microglia, tumor microenvironment, immunotherapy

## Abstract

Glioma is a mixed solid tumor composed of neoplastic and non-neoplastic components. Glioma-associated macrophages and microglia (GAMs) are crucial elements of the glioma tumor microenvironment (TME), regulating tumor growth, invasion, and recurrence. GAMs are also profoundly influenced by glioma cells. Recent studies have revealed the intricate relationship between TME and GAMs. In this updated review, we provide an overview of the interaction between glioma TME and GAMs based on previous studies. We also summarize a series of immunotherapies targeting GAMs, including clinical trials and preclinical studies. Specifically, we discuss the origin of microglia in the central nervous system and the recruitment of GAMs in the glioma background. We also cover the mechanisms through which GAMs regulate various processes associated with glioma development, such as invasiveness, angiogenesis, immunosuppression, recurrence, etc. Overall, GAMs play a significant role in the tumor biology of glioma, and a better understanding of the interaction between GAMs and glioma could catalyze the development of new and effective immunotherapies for this deadly malignancy.

## 1 Introduction

Glioblastoma (GBM), also known as IV grade glioma, is the most common and malignant neoplasm in the adult central nervous system (CNS). Despite surgical resection, targeted radiotherapy, combined chemotherapy, and newer developed treatments like tumor-treating fields (TTF), the prognosis of GBM patients remains very poor (1). The median survival time of adult GBM patients is less than 15 months with traditional treatment and less than 20 months with TTF treatment (1, 2). This poor prognosis is attributed to the highly aggressive nature of GBM, which is characterized by progressive growth, diffuse invasiveness, and frequent resistance to chemotherapy (1). Besides the inability to completely resect the tumor body due to invasive growth of the tumor and cell heterogeneity of the GBM stem cells (GSCs) in the glioma, tumor-associated immune cells significantly contribute to the high malignancy and proliferation of GBM (3). These immune cells as well as other noncancerous cells such as normal and reactive astrocytes, GSCs, fibroblasts, vascular pericytes, and endothelial cells form the tumor microenvironment (TME), which assists tumor development by releasing various cytokines, chemokines, growth factors, and other hormones (4–6). In recent years, researchers have conducted comprehensive studies on TME using genomics, proteomics, and other technologies.

Although many types of immune cells within the lymphoid lineages have been detected in the TME, glioma-associated macrophages and microglia (GAMs) are the predominant immune population in the solid GBM, comprising up to one-third of the tumor mass (7–9). This is primarily due to the critical role resident macrophages and microglia play in the innate immunity of the brain, an organ known for its immune privilege (9). Recently, T-cell-based immunotherapies have demonstrated curative potential in several non-intracranial malignancies, such as B-cell acute lymphoblastic leukemia and advanced renal-cell carcinoma (10–12). However, although T-cells can infiltrate the tumor body and surrounding areas of the GBM, the application of T-cell rejuvenation strategies for GBM has produced contradictory results due to the low number of T-cells and the lack of key stimulators (13). Although immune checkpoint blockade regimens including cytotoxic T lymphocyte antigen 4 and programmed cell death 1 (PD-1) have been employed, they have not demonstrated a significant improvement in the survival time of GBM patients (13, 14). Dominant GAMs in GBM mediate low levels of proinflammatory factors and a lack of key T-cell costimulatory factors, leading to a weak response state of T-cells in GBM (15). Additionally, the presence of GAMs in GBM is a known indicator of poor prognosis (8, 9), as GAMs are biased toward M2 polarization, which promotes heterogeneous differentiation, diffusion growth, and tumor recurrence (7, 16). Nonetheless, GAMs possess a feature of plasticity, which highlights the potential of developing new therapeutic methods based on their metabolism and genome regulation. In this review, we will summarize the origin of GAMs, their relationship with GBM, and recently developed immunotherapies targeting GAMs.

## 2 The origin, physiological function, and subtype transformation of microglia and macrophages in the CNS

Microglia are a critical innate immune component in the CNS and represent 10-15% of all glial cells (17). Due to their phagocytic activity and origin with peripheral myeloid cells, microglia are considered the tissue resident macrophages of the CNS (17, 18). In the physiological state, the number of macrophages in the CNS is much less than that of microglia (19), and they are mainly distributed in the perivascular space, meninges, and organs surrounding the ventricles and choroid plexus, and are rare in brain parenchyma (19). These macrophage populations are highly heterogeneous and can be replaced to a certain extent. In pathological conditions such as injury, infection, degenerative diseases, and tumors, microglia, and macrophages display different polarization states and express specific markers (19, 20).

### 2.1 Microglia

Microglia derive from hematopoietic precursor cells of the yolk sac during early embryonic development (17, 21), and Runt related transcription factor-1 (Runx-1) and colony stimulating factor-1(CSF-1) play critical roles in their development (21). Due to their powerful phagocytic function, microglia can engulf abnormal entities in the CNS, such as tumor cells, necrotic cell fragments, and pathogens (21). In addition, microglia perform immune regulatory functions, interact with neurons and glial cells, and promote angiogenesis (22). Therefore, microglia not only effectively respond to CNS damage, infection, and mutation, but also play a critical role in the development and homeostatic maintenance of brain (21, 22). Microglia rapidly shift from a ramified resting state to an amoeboid-like activated state and release reactive oxygen species (ROS), proinflammatory cytokines, and chemokines in response to exogenous and endogenous stimuli, such as infection and injury (23). This effect is mainly achieved through pattern recognition receptors (PRRs) expressed in microglia, which recognize pathogen-associated molecular patterns (PAMPs) and damage-associated molecular patterns (DAMPs) (24, 25). Recent studies have also shown that microglia play an important role in synaptic formation in the mature brain (26), serving as intermediates for information exchange through hemichannels and gap junctions with neurons and other glial cells (26). Moreover, during the development of primary tumors, activated microglia can kill tumor cells by secreting proinflammatory cytokines and other factors (9). However, with tumor progression, the phenotype and function of microglia change, causing damage to normal neural structures and promoting tumor growth (9, 27). This topic will be discussed in detail later.

Due to the lack of reliable *in vivo* and *in vitro* experimental models of microglia, the investigation of microglia in the context of tumors remains limited. Alongside primary microglia cells extracted from experimental animals or human brains, immobilized murine (BV-2) and human (HMO6) microglia cell lines have been developed (28). Nevertheless, it is crucial to note that there exist differences between the primary brain-derived microglia and the immortalized BV-2 cell line at the transcriptional level (29). Recent advancements in flow sorting technology and transcriptome sequencing, several specific marker molecules of primary microglia have been identified. For example, some transcription factors, including Rhox5, E2f6, Hoxc6, and Ppargc1b are exclusively expressed in microglia (30, 31). Furthermore, certain membrane proteins, Lrp8 and Lpcat3, which are associated with lipid metabolism, and ion transporters, like Slco4a1 and Slc30a5, are unique to microglia compared with macrophages (30, 31). Potential markers that distinguish CNS-derived microglia and BMDMs are systematically summarized in **Table 1**.

**Table 1:** Markers for different polarization types of GAMs.

Biomarkers	Molecular type	M1/M2	Macrophages/Microglia	Remarks
**IBA1**	Cytoskeleton binding protein	NA	Macrophages and Microglia	Higher identification value for microglia in CNS
**F4/80**	Surface glycoprotein	NA	Macrophages and Microglia	Mouse-specific; Multiple macrophage lineage cells
**CD68**	Glycoprotein	NA	Macrophages and Microglia	NA
**TMEM119**	Transmembrane protein	NA	Microglia	Reliable CNS-resident microglia marker
**CD11b^+^/CD45^low^**	Transmembrane protein	NA	Microglia	Not specific because of the influence of inflammation in glioma
**CD11b^+^/CD45^high^**	Transmembrane protein	NA	BMDM	Not specific because of the influence of inflammation in glioma
**Rhox5, E2f6, Hoxc6, Ppargc1b, etc.**	Transcription factors	NA	Microglia	Transcriptome study on the mouse microglia
**Slco4a1, Slc30a5, and Mcoln3**	Membrane proteins	NA	Microglia	Transcriptome study on the mouse microglia
**CD40, CD74, CD86 and MHC II**	Glycoprotein	M1	NA	CD80^high^/CD86^high^ is specific for M1
**iNOS/NO**	Metabolic enzyme	M1	NA	NA
**CD14, CD163, CD204/206**	Glycoprotein	M2	NA	The specificity of some markers is not clear
**ARG1**	Membrane proteins	M2	NA	NA

IBA1, ionized calcium-binding adapter molecule 1; TMEM119, transmembrane protein 119; BMDM, bone marrow derived macrophage; MHC II, histocompatibility complex II; ARG1, Argininase 1.

NA means "Not applicable".

### 2.2 Conundrums in the identification of GAMs

Brain-infiltrating bone marrow-derived macrophages (BMDMs) originate from hematopoietic stem cells (32) and infiltrate the brain parenchyma in large numbers after the development of GBM due to the destruction of blood-brain barrier and release of multiple chemokines by the tumor tissue (33, 34). They are mainly located in perivascular and necrotic regions to address the ischemic regions of the tumor (33). Although microglia and infiltrating BMDMs have distinct origins, they perform similar immune regulatory functions and express several common markers, such as ionized calcium-binding adapter molecule 1 (IBA1), CD11b, CD68, CX3C chemokine receptor 1 (CX3CR1) (9, 35, 36). Among these markers, IBA-1 and CX3CR1 were thought to be specific to microglia, however, subsequent studies confirmed that they were also expressed by BMDMs (22, 37). In contrast, major histocompatibility complex (MHC) class II and subsequent Sall1 are specific to microglia and can be used to differentiate between microglia and macrophages in the brain parenchyma (22, 38). In addition, transmembrane protein 119 (TMEM119) is a recently discovered marker with high specificity for microglia that can distinguish microglia and macrophages in both human and mouse models (35, 39). CD49D is specifically expressed by BMDMs infiltrating malignant brain tumors in mice and humans, but not by microglia (33). C-C chemokine receptor-2 (CCR2) was previously considered as another specific marker to differentiate macrophages in TME, but subsequent studies found that CCR2 is also expressed by microglia, particularly those activated by interaction with glioma cells (40). Previously, CD45 has been used to distinguish between resident microglia (CD45^low^) and infiltrating macrophages (CD45^high^) (41), and in combination with CD11b, microglia and macrophages in rodent and human GBM can be defined as CD11b^+^/CD45^low^ and CD11b^+^/CD45^high^ populations, respectively (42). However, CD45 expression increases in microglia activated in the TME, which adds complicates the identification of microglia and macrophages in tumors (42). To date, no lineage-specific markers have been developed to accurately distinguish these two cell populations, which has made it challenging to assess the specific role of each cell type in tumor development.

### 2.3 The subtypes of microglia and macrophage in GBM

Mills et al. were the first to define macrophages as M1 polarization type for pro-inflammatory (antitumor) and M2 polarization type for anti-inflammatory (protumor), referring to the dichotomy of CD4^+^ T cell function into T helper (Th) 1 and Th2 lineages (43). Ponomarev et al. subsequently verified the polarization of microglia in the rodent CNS (44). Numerous CNS pathological events, such as tumor occurrence, injury, microbial infection, and degenerative diseases, can cause the polarization of microglia/macrophages (33, 45–47). For instance, microglia, which account for the majority of GBM, can be polarized into the M1 phenotype under the stimulation of lipopolysaccharides (LPS) or interferon-*γ* (IFN-γ) (13). M1 type microglia reactively express co-stimulatory molecules such as tumor necrosis factor-alpha (TNF-α), interleukin-6 (IL-6), and chemokine (C-X-C motif) ligand 10 (CXCL10), and high-level of MHC class II, which endows them with the function of antigen presentation (13). In the context of glioma, M1 microglia play an anti-tumorigenic role through antigen presentation mediated adaptive immunity response (phagocytosis of tumorigenic cells) and secretion of proinflammatory factors (8, 48). Inflammatory factors such as TNF-α further recruit peripheral macrophages into the tumor (49). M1 microglia/macrophages have the classic activated phenotype, and their activated receptors and functions are relatively clear. The polarization of M2 microglia and macrophages is strictly regulated and presents a highly dynamic state, often followed by M1 polarization (49, 50). Type II inflammatory factors such as IL-4, IL-10, and IL-13 generally mediate M2 polarization, which can prevent further tissue damage by secreting multiple anti-inflammatory factors to downregulate the inflammatory response (51, 52). In the context of glioma, M2 microglia promote tumor development (53). M2-polarized microglia/macrophages are differentiated by the co-expression of surface markers such as CD163, CD204, CD206 as well as arginase (53). We have a systematic list of markers for M1 and M2 microglia/macrophages in **Table 1**. M2 microglia/macrophages can be further divided into several subtypes (M2a, M2b, M2c) based on their expression of different transcription factors, effector functions, and secretion of cytokines and chemokines (54). M2a and M2b microglia/macrophages are responsible for Th2 activation and immunoregulation (55, 56), while the M2c subtype attenuates inflammatory response, promotes matrix deposition and tissue remodeling, significantly promoting tumor development in the neoplastic context (57–59).

Recent single-cell sequencing studies on GBM suggest that individual cells can express genes promoting inflammation (M1) and genes promoting immunosuppression (M2) (60, 61). These findings challenge the precision of the conventional M1/M2 typing of GBMs and suggest that the polarization may be a highly continuous process in the context of GBM, where GAMs are highly plastic (8). Furthermore, there are likely unclearly defined GAM subtypes having important specific functions in human and rodent GBM. For example, the nonpolarized M0 subtype may have been overlooked to some extent, even though it represents a weakened M2 subtype (62). Accurate classification of GAMs is critical for guiding the development of therapeutic drugs, but it is challenging to establish a systematic microglia/macrophage classification system. Thus, the classical subtype classification model still holds important reference value.

## 3 Interaction between GAMs and GBM cells

**Figure 1:**
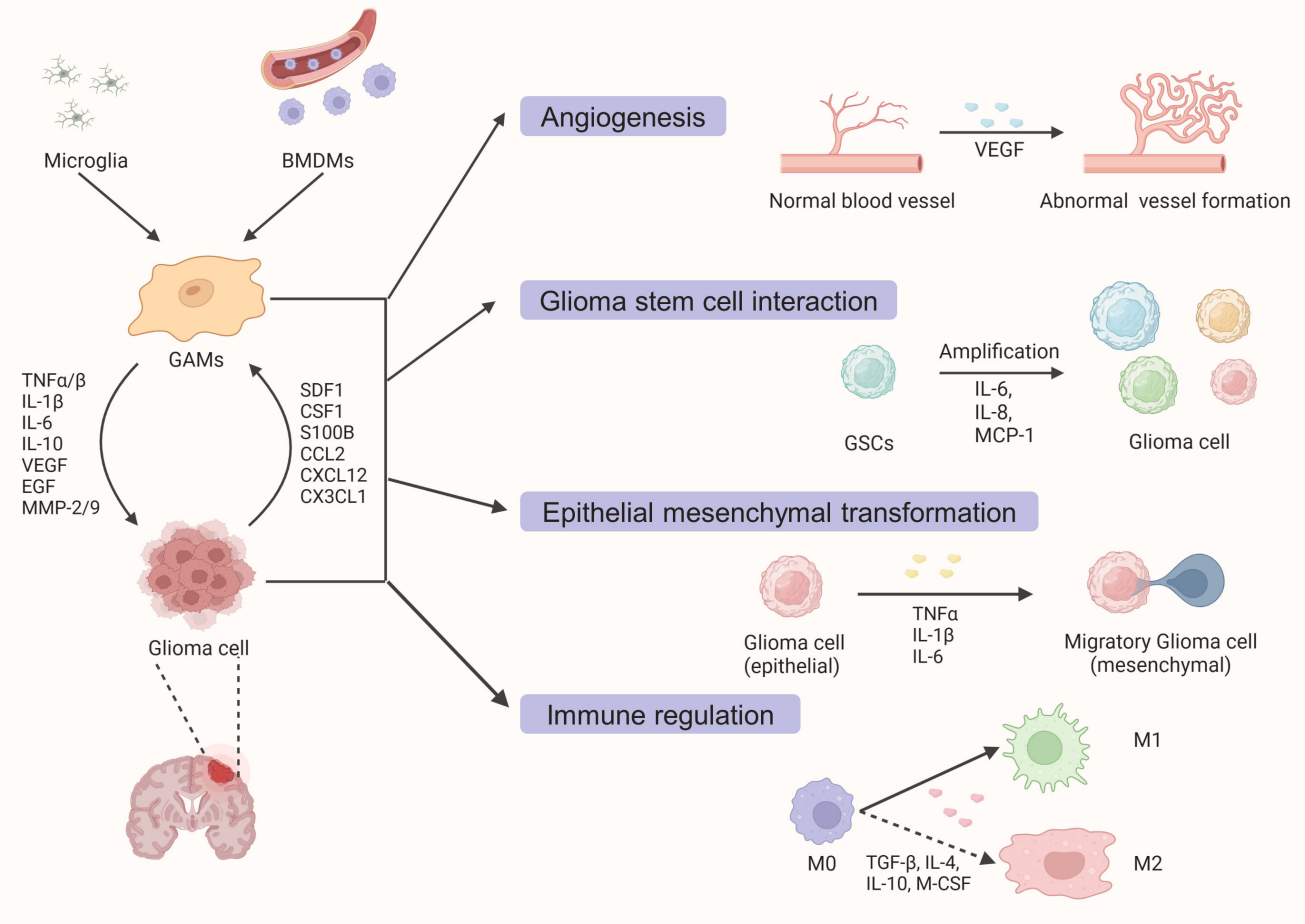
A schematic diagram outlining the interaction between glioma cells and glioma-associated macrophages and microglia (GAMs). Under the action of various cytokines and chemokines secreted by tumor cells, resident microglia, and peripheral blood-derived macrophages are recruited into the tumor parenchyma. Activated GAMs interact with tumor cells to promote glioma growth and invasion through various mechanisms such as angiogenesis promotion, GSCs proliferation, epithelial-mesenchymal transformation, and tumorigenic immune regulation.

Glioma-associated microglia/macrophages (GAMs) are not passive bystanders in the tumor microenvironment, but actively evolve with tumors through feedback and feedforward mechanisms (**Figure 1**). While most of the mechanisms involved are feedback regulation mechanisms, some feedforward mechanisms also play important roles in the recruitment of GAMs by glioma cells. For instance, glioma cells can secrete versican, which promotes the activation of microglia through the Toll-like receptor 2 (TLR2) signaling pathway (63, 64). Some researchers even propose the hypothesis that the immune system does not recognize malignant tumor cells as invaders in the CNS, but rather helps them infiltrate and grow (65). These effects are largely due to the recruitment of microglia/macrophages by tumor cells. After being polarized into a tumor-promoting M2 phenotype, GAMs assist tumor development by promoting invasive growth, inducing angiogenesis, interacting with GSCs, and mediating the formation of an inhibitory TME (9).

### 3.1 The recruitment of GAMs and induction of M2 polarization by glioma cells

A large number of previous studies have demonstrated that glioma cells secrete different chemokines, which serve as chemoattractants and mediate the recruitment of GAMs. These chemokines include monocyte chemoattractant protein (MCP)-1 (alternative name: C-C motif ligand 2 (CCL2)) (66), MCP-3 (67), stroma-derived factor (SDF)-1 (alternative name: CXCL12) (68), lysyl oxidase (LOX) (69), macrophage colony-stimulating factor (M-CSF) (70), and glial cell–derived neurotrophic factor (GDNF) (71). The role of MCP-1 in GAMs has been verified by various *in vivo* and *in vitro* studies, where the MCP-1 expression level is highly correlated with the grade of glioma (66). A recent study indicated that the cooperation between β- Catenin and MCP-1 may be responsible for the rapid and highly heterogeneous growth of Isocitrate dehydrogenase wildtype GBM (72). M-CSF is another important chemokine, which not only promotes the mobility of GAMs but also mediates the M2 polarization of GAMs (73). Gliomas having a phosphatase and tensin homolog (PTEN) deletion highly express LOX, which activates the β 1 integral/proline-rich tyrosine kinase 2 pathway in GAMs, aiding their recruitment (69).

In recent years, researchers have suggested alternative factors that might impact the recruitment of GAMs and M2 polarization, such as peritumoral hypoxia microenvironment-induced factors and GSCs (74). Hypoxia may be the most critical regulatory factor for the recruitment of GAMs, as a large portion of the glioma is hypoxic and harbors large numbers of M2 polarization-type GAMs. Recent studies suggest that hypoxia may affect the expression pattern of chemokines, especially in perivascular niches. For example, Guo et al. revealed that TGF-α mediated upregulation of periostin (POSTN) in the peritumoral region of the glioma significantly promotes the recruitment of GAMs (75). Another recent study showed that arginine methyltransferases (PRMTs) enhance the recruitment of GAMs by enhancing hypoxia-inducible factor-1 mediated hypoxia, thus promoting glioma progression (76). Specific chemokines secreted by GSCs may recruit certain GAMs subtypes. For example, POSTN secreted by GSCs specifically recruits M2 GAMs through integrin αvβ3 signaling pathway (53). In addition, some cytokines with undefined functions have also been reported to participate in the recruitment of GAMs. For example, dual function cytokine IL-33, secreted at the nucleus of gliomas, was recently demonstrated to recruit and activate circulating and resident innate immune cells (77).

### 3.2 GAMs promote glioma invasion and angiogenesis

The tumor-promoting effects of GAMs have been demonstrated in organotypic brain tumor-slice cultures as well as several *in vivo* models (78–80). Co-culture of glioma cells with microglia extracted from mouse brain significantly increased glioma cell migration, while the deprivation of microglia significantly promoted tumor growth and invasion (78, 79). A range of anti-inflammatory and pro-tumoral factors secreted by GAMs have been identified as playing important roles in glioma cell invasion, including transforming growth factor beta (TGF-β), epidermal growth factor (EGF), IL-6, IL-1β, stress-inducible protein-1 (STI-1), matrix metallopeptidase-2 (MMP-2), and MMP-9 (27). Among these, MMP-2 and MMP-9 are important effector molecules that enhance glioma cell invasiveness by breaking down extracellular matrix (ECM) components such as collagen and elastin. MMP-2 expression is positively correlated with glioma invasiveness and poor patient prognosis (81). TGF-β, an inhibitory immune regulatory factor, is the most widely explored factor in the mechanism of invasive growth of glioma. TGF- β released from GAMs has been demonstrated to promote the secretion of MMP-2 and MMP-9, resulting in enhanced GBM invasiveness (82). Recent studies have revealed that MMP-2 may be an important downstream molecule of CCL5 in promoting the migratory and invasive activities of GBM (83). MMP-9 has also been reported to be an effector molecule in the tumorigenic infiltration of GBM mediated by GAMs (84). GBM released CCL2 has been reported to upregulate IL-6 expression, which is responsible for GBM invasiveness in a TLR4-dependent fashion (85). The expression level of IL-6 is highly correlated with the pathologies of GBM patients (86). EGF is considered a promoter of GAM-mediated tumor invasiveness, as it is not detectable in the supernatant or cell lysate of glioma cells cultured separately (70). EGF primarily binds to the surface of GBMs to promote tumor invasion. Amplification of the EGFR gene and its truncation mutant are present in over half of primary GBM and are indicative of highly aggressive tumors (87). It should be noted that the regulatory pathways discussed above do not strictly follow the effect of GAMs on GBM in the context of GBM. Many regulatory factors can also be released by tumor cells and influence GAMs.

Angiogenesis is a critical factor in the growth and progression of glioma. GAMs extracted from GL261 gliomas have been shown to release a plethora of proangiogenic molecules, including vascular endothelial growth factor (VEGF) and CXCL2 (88). Depletion of resident microglia significantly reduces tumor vessel count, and a high spatial correlation between GAMs and tumor neovascularization has been reported (88). For instance, specific GAMs that are absent in normal brain tissue were directly detected in the perivascular niche (merged with CD31^+^ vessels) (89). Furthermore, studies have not only verified the direct contact effect of GAMs with blood vessels around and inside the tumor using allografted mice but have also detected elevated levels of various angiogenesis-inducing factors, such as VEGF, VEGFR1, CCR2, CXCR4, CCL2/5, and CXCL2/10/14 (14, 90–93). Recent research has confirmed the strong correlation between GAMs and tumor blood vessels in GBM patients and observed the restructuring of the blood vessel architecture, indicating the potential of anti-angiogenic therapy in the treatment of GBM (88). Consistent with this, VEGF receptor (VEGFR) blocker Sunitinib and VEGF inhibitor Bevacizumab have been shown to promote survival by reducing tumor angiogenesis in the mouse GBM model (94).

### 3.3 The role of GAMs in shaping immune homeostasis and promoting an immunosuppressive TME

The immune-escape mechanism of GBM relies on two main factors: the intrinsic characteristics of neoplastic cells and the immunosuppressive TME mediated by GAMs. Glioma cells are difficult to identify by the immune due to their downregulation of human leukocyte antigen (HLA) molecules that cover the surface specific tumor antigen molecules (95). However, the immunosuppressive TME has been recognized as a more significant contributor to promoting tumor immune evasion, facilitating its growth, invasion, and recurrence (78, 79). GAMs, especially the type M2 subtype, are considered major contributors to TME due to their secretion of type II immune factors such as TGF-β, IL-4, and IL-10 (4, 6). These cytokines upregulate transcription factors such as signal transducer and activator of transcription 3 (STAT3) in glioma cells, which subsequently triggers tumorigenic immune responses (27). STAT3 plays a vital role in the interaction between tumor cells and GAMs, and its activation significantly enhances tumor-promoting immune regulation while inhibiting tumor-killing immune response (96, 97). Several studies have demonstrated that glioma cells secrete S100 calcium binding protein B (S100B), which promotes M2 polarization of GAMs through the receptor for advanced glycation end products (RAGE)-STAT3 signaling pathway (98). Additionally, other growth factors secreted by type M2 GAMs, such as platelet derived growth factor (PDGF), EGF, and fibroblast growth factor-2 (FGF-2), have been reported to activate STAT3, promoting GBM progression (99–101). *In vitro* studies have shown that conditioned medium derived from glioma cells can activate the STAT3 pathway of microglia, promoting their polarization towards the M2 subtype and secretion of IL-6 and IL-10 (102).

A recent study found that blocking the breakdown of interferon gamma inducible protein 16 (IFI16) with drugs can activate the STAT3 signaling pathway (103). This pathway is crucial for GBM progression because it promotes cell proliferation, invasion, and regulation of the TME. Moreover, high levels of phosphorylated-STAT3 (pSTAT3) are associated with more severe gliomas and poorer patient outcomes (104–106). This is likely because pSTAT3 can suppress the immune system, leading to greater resistance to standard cancer treatments and an increased risk of tumor recurrence (105, 106).

### 3.4 Interactions between GAMs and GSCs

GCSs are a group of cells that are resistant to chemotherapy and contribute to tumor growth and recurrence (107–109). They share stem cell-like characteristics, which enable them to regenerate and differentiate into different cell types (108, 109). This allows them to continuously generate new tumor cells at the site of the tumor. GSCs are similar to NSCs in terms of their phenotypic characteristics. For example, they can form neurospheres *in vitro* and express common markers such as nestin, Sox2, and Musashi-1 (110, 111).

GSCs have low mitotic activity, which makes them resistant to therapies directed at actively dividing cells, such as temozolomide (TMZ)-based chemotherapy, and enables them to cause tumor recurrence (112, 113). They also have low expression of molecules that present tumor antigens to CD8^+^ T cells, and immune checkpoint pathways are often activated in glioma cells (114). These mechanisms help tumor cells evade immune surveillance. GSCs interact with various components in TME to maintain their drug resistance and tumorigenicity. They are located in specific niches where they are protected from therapy exposure and niche-specific factors (95, 115, 116). GAMs mainly accumulate in perivascular and perinecrotic hypoxic niches, where they initiate interactions with SGCs to affect glioma prognosis (74, 95). GSCs secrete chemokines (such as VEGF, CCL2, CCL5, and CCL7), to recruit GAMs to tumor mass and induce M2 type polarization of GAMs, aiding their transformation and proliferation by building a tumorigenic TME. Apart from GSCs-secreted chemokines, GSCs also exhibit higher levels of neurotensin than those in non-GSC glioma cells (27, 117). *In vitro* studies have shown that GSCs recruit GAMs more effectively than glioma cell lines. Moreover, a positive correlation between GAMs and GSCs has also been reported (118). These findings emphasize the importance of GSCs for GAMs recruitment.

Conversely, GAMs have been shown to play a role in promoting GSCs (119–122). Research by Wang et al. demonstrated that IL-6, which is secreted by microglia, can function as a growth factor for GSCs and enhance their biological function (122). It is important to note that only GAMs, not the naïve resident microglia, can promote GSC amplification (123). Interestingly, microglia from healthy individuals can even suppress glioma growth by expressing IL-8 and MCP-1 (123). In general, GAMs and GSCs interact extensively in glioma, jointly regulating tumor progression, recurrence, and drug resistance.

## 4 GAMs-targeted glioma-immunotherapy

The rapid development of new tumor treatment technologies and a deeper understanding of CNS have led to significant progress in immunotherapy for glioma (124). Some of these advancements have already undergone clinical trials, such as dendritic cell vaccine (DC Vax-L), chimeric antigen receptor T cell therapy (CAR-T), and immune checkpoint inhibitors (1–3, 125, 126). As highlighted earlier, GAMs play a critical role in glioma progression, invasion, recurrence, and drug resistance, making them a promising intervention target for glioma immunotherapy. The basic concept of GAMs targeted immunotherapy involves inhibiting the recruitment and infiltration of GAMs, inhibiting the polarization of the M2 phenotype, or eliminating the M2 phenotype and promoting the transformation of the M2 and M0 phenotypes into M1 through reprogramming and other means, thereby restoring their tumor-killing effect. **Table 2** summarizes some representative immunotherapy approaches targeting GAMs.

Table 2Therapeutic drugs targeting GAMs in glioma.

**Table:** 

Clinical trials					
Drug name	Study phase	Action target	Potential mechanism of action	Tumor Types	Reference
**Minocycline**	I	Inhibit microglia	Suppression of microglial activation-mediated radiation resistance	HGG	(127)
**Pexidartinib (PLX3397)**	II and I/IIb	Inhibit CSF-1R	Elimination of GAMs	rGBM, pGBM	(128)
**Emactuzumab**	I	Inhibit CSF-1R	Suppression of GAMs polarization and inhibition of glioma progression	GBM	(129)
**IL-12**	II	N/A	Immune checkpoint blockade with controlled IL-12 gene therapy	rGBM	(130)
**CpG-ODN**	II	N/A	M1 polarization of GAMs (no effect on survival of patients)	*De novo* GBM-A	(131)
**Plerixafor**	I/II	Inhibit SDF-1	Suppression of GAM infiltration by inhibition of chemotaxis	HGG	(132, 133)
**WP1066**	II	Inhibit STAT3	Promotion of M1 polarization of GAMs by blocking STAT3	GBM, glioma	(134)

**Table:** 

Preclinical trials					
Drug name	Drug type	Action target	Potential mechanism of action	Tumor Types/Models	Reference
**Minocycline**	antibiotic	Microglia	Reduction of microglial activation mediated MMP-9 and TLR2	Glioma/*in vivo*	(64)
**Amphotericin B**	antibiotic	Microglia	Promotion of the activation of M1 GAMs	Glioma/*in vitro*	(123)
**Pexidartinib (PLX3397)**	Antibody	Inhibit CSF-1R	Inhibition of GAM recruitment	GBM /*in vivo and in vitro*	(135, 136)
**AFS98 and BLZ945**	CSF-1R inhibitor	Inhibit CSF-1R	Upregulation of M2 GAMs markers (Use in combination with other drugs)	GBM/*in vitro*	(137)
**IL-12**	Cytokine	N/A	GAMs induced apoptosis of GBM cells *via* TRAIL-DR4/5	GBM/*in vitro*	(138)
**Lps or IFN-γ**	Stimulus and Cytokine	N/A	GAMs induced apoptosis of GBM cells	GBM/*in vivo*	(139, 140)
**CpG-ODN**	oligodeoxynucleotides	N/A	M1 polarization of GAMs and Type 1 inflammatory reaction	GBM/*in vivo*	(141)
**WP1066**	STAT3 inhibitor	Inhibit STAT3	Inhibition of STAT3 enriched K27M-mutant cells	H3K27M-mutant DMG /*in vivo*	(142)
**NAcp@CD47**	nanocapsule	Inhibit CD47	Enhancement of antitumor immunogenicity by inhibition of CD47/SIRPa	pGBM/*in vivo*	(143)

HGG, high grade glioma; CSF-1R, colony stimulating factor 1 receptor; GAMs, glioma-associated macrophage and microglia; rGBM, recurrent GBM; pGBM, primary GBM; IL-12, interleukin-12; SDF-1, stroma-derived factor-1; STAT3, signal transducer and activator of transcription 3; MMP-9, matrix metalloprotein-9; TLR2, Toll-like receptors-2; DMG, diffuse midline glioma; TRAIL, tumor necrosis factor-related apoptosis-inducing ligand; DR4/5, death receptor 4/5; SIRPa, signal regulatory protein-alpha.

NA means "Not applicable".

Several antibiotics and inflammatory cytokines/substances have been reported to regulate the origin and activation of GAMs, particularly microglia, to exert effective anti-tumor effects. For instance, minocycline, a broad-spectrum tetracycline antibiotic, has been shown to reduce microglia activation and infiltration by mitigating matrix degradation, thereby exhibiting anti-tumor effects (64). Similarly, several preclinical studies have demonstrated that amphotericin B, a polyene antifungal drug, can inhibit glioma development by activating M1 GAMs (123). Researchers found that treatment with amphotericin B promotes M1 polarization of GAMs by inducing TLR signaling pathways and significantly prolongs the survival time in the mouse model of glioma (123, 144). Furthermore, IFN-γ, IL-12, LPS, and oligodeoxynucleotides containing CpG motifs (CpGODN) have been shown to increase the M1 polarization of GAMs, leading to the elimination of tumor progression *in vivo (* 138–141). Recently, a phase II clinical trial for recurrent GBM has been conducted using combined immunotherapy including IL-12 gene-regulated therapy (130).

CSF-1 (M-CSF)/CSF-1R signal is considered a key factor in GAMs recruitment and M2 polarization. Therefore, researchers have explored drugs targeting this pathway. Treatment with the anti-CSF-1R antibody pexidartinib (PLX3397) significantly reduces GL261-associated GAM infiltration and inhibits M2 polarization, thereby inhibiting glioma growth (135). PLX3397 treatment significantly prolonged the survival time of mice with glioma. However, in the phase II clinical trial, PLX3397 as a monotherapy failed to affect recurrent GBM patients (128), mainly due to drug resistance. This highlights the need for combination therapy. Similarly, the application of another anti-CSF-1R antibody, Emactuzumab (RG7155), also failed to achieve the therapeutic effect, likely due to reactive overproduction of IL-4 by glioma cells (129). In addition, treatment with CSF1R inhibitors such as AFS98 and BLZ945 increased the expression of M2 GAM markers, but they did not inhibit glioma growth as single agents (137).

SDF-1 (CXCL12) is another factor crucial in GAMs recruitment, especially under normoxic conditions. The Food and Drug Administration (FDA)-approved drug for the treatment of multiple myeloma and lymphoma, SDF-1 inhibitor Plerixafor, is currently being evaluated in two clinical trials for its potential therapeutic effect against glioma (132, 133). As mentioned earlier, STAT3 is the central transcription factor mediating the M2 polarization of GAMs. WP1066 has been reported to inhibit the growth and recurrence of glioma by suppressing the protein synthesis of STAT3 (142). John de Groot et al., in their recently concluded Phase I clinical trial of WP1066, have determined the maximum allowable dose of WP1066 to be 8 mg/kg (134).

Microglia/macrophages exhibit strong phagocytic activity. However, in glioma, the phagocytosis of GAMs is greatly reduced, due to the high expression of CD47 along with signal regulatory protein alpha (SIRPa) on the surface of GAMs (145, 146). Therefore, a therapeutic approach involving a blockade of this signaling pathway using anti CD47 antibody to restore the phagocytic activity of GAMs has been proposed (145, 146). Recently, Zhou et al. have developed a novel nano-capsule loaded with anti-CD47 antibodies, which could prove to be useful in testing the anti-tumor potential of this therapeutic approach (143).

Recent studies have shown that olfactomedin Like 3 (OLFML3) exhibits an anti-glioma effect by regulating GAMs infiltration under the influence of the biological clock, and a positive correlation between the survival of GBM patients and the expression level of OLFML3 has been reported (147).

## 5 Conclusion

Although researchers have extensively studied the origin, evolution, recruitment, and other mechanisms of microglia/macrophages, the role of GAMs in TME is still poorly understood. This is because the underlying mechanisms do not act in isolation in the tumor context. Glioma cells and various components of the TME form a complex interaction network. Although novel therapies are being developed, GBM recurrence remains a challenge. A deeper understanding of various aspects of GAM biology may provide useful insights for the development of effective strategies for glioma immunotherapy. Moreover, understanding the role of the TME in the development and evolution of tumorigenic cells from a broader perspective, rather than limiting the focus to glioma cells and their transformation, would provide a holistic perspective. For example, the niche of GSCs, which influences tumor diversity and drug resistance, is of great research significance. In the future, the combined treatment with chemotherapy drugs as well as monotherapies involving immunotherapeutic strategies may prove useful in improving the life quality of glioma patients.

## Author contributions

Conceived and designed the review: CYX. Wrote and revise the paper: CL and NW. All authors contributed to the article and approved the submitted version.
